# Supplementary material for: Cost impact of chronic care management services in a large multi-specialty practice: A pragmatic outcomes study
Source: PLoS One. 2026 Oct 30;21(10):e0345422. doi: 10.1371/journal.pone.0345422 (PMC13722291; doi:10.1371/journal.pone.0345422)
Supplement: Supplementary file 1 [file pone.0345422.s001.pdf]

ScreenIT Report

This is an automated report for 10.64898+2026.03.07.26347834.pdf. This report was solicited by the PLOS One editorial team and provided by ScreenIT.

ScreenIT is an independent group of scientists developing automated tools that analyze academic papers. A set of automated tools screened your submitted manuscript and provided the report below. Each tool was created by your academic colleagues with the goal of helping authors.

The tools look for factors that are important for transparency, rigor and reproducibility, and we hope that the report might help you to improve reporting in your manuscript. Within the report you will find links to more information about the items that the tools check. These links include helpful papers, websites, or videos that explain why the item is important.

While our screening tools aim to improve and maintain quality standards they may, on occasion, miss nuances specific to your study type or flag something incorrectly. Each tool has limitations that are described on the ScreenIT website. The tools screen the main file for the paper; they are not able to screen supplements stored in separate files.

Please note that the Academic Editor had access to these comments while making a decision on your manuscript. The Academic Editor may ask that issues flagged in this report be addressed.

If you would like to provide feedback on the ScreenIT tool, please email the team at ScreenIt@bih-charite.de. If you have questions or concerns about the review process, please contact the PLOS One office at plosone@plos.org.

How to read the report:

- no action required

? - review for potential issues

! - a critical issue was found by a tool

n/a - may not be applicable to your paper

- more information about this item can be obtained using this link

Rigor

✓	Ethics	Consent: "Verbal consent was documented in the patient's medical record, and a follow-up letter confirming enrollment was sent to each participant."<br>IRB: "The study was determined to be exempt from institutional review board oversight under federal regulations for quality improvement activities."
✓	Inclusion and Exclusion Criteria	"Inclusion criteria required individuals to have at least two chronic conditions, as defined by ICD-10 codes corresponding to the Healthcare Cost and Utilization Project (HCUP)/Chronic"
?	Flow charts and attrition	We did not find a study flow chart of excluded observations. We strongly recommend using flow charts because they provide an overview of the study design and more information about attrition. If you included a study flow chart in your supplemental files, we apologize for missing this. Our tool is not able to screen separate supplemental files.<br><br>Sentence about attrition: "No patient identifiers were included in the analytic dataset."
✓	Sex as a biological variable	"On average, the treatment group was 2% older than the comparison group (mean age 74.5 years vs. 72.8 years) and included 10% more female beneficiaries (62% vs. 56%)."
✓	Subject Demographics	Age: "On average, the treatment group was 2% older than the comparison group (mean age 74.5 years vs. 72.8 years) and included 10% more female beneficiaries (62% vs. 56%)."
?	Randomization	Not detected. If you performed an experimental study, please specify whether participants, animals or samples were randomly assigned to treatments or groups, and specify the randomization procedure.
?	Blinding	Not detected. Please specify whether blinding was used at various phases of the experiment (e.g., blinding of patients,

		caregivers, outcome assessments, data analysis).
?	Power Analysis	Not detected. Please state how you determined the sample size for the study. If you performed a power or sample size calculation, please provide details.
n/a	Replication	not required.

Generated by Barzooka (flow charts), SciScore (everything else).
SciScore only screens the text of the methods section; we apologize if the tool has missed items reported elsewhere in the paper or supplements.

Resources

Persistent unique identifiers for research resources, RRIDs, can be used to track these key resources though the literature. They are requested or required in many journals, but even if your journal does not require them, we strongly encourage the use of these for the readers of papers who wish to easily track down the key materials like antibodies, software tools, or mice.

Software and Algorithms	"For example, in patients with hypertension, plans incorporated recommendations from the American Heart Association, behavioral support for physical activity, medication adherence counseling, education on medication side effects, and dietary modifications consistent with the DASH (Dietary Approaches to Stop Hypertension) diet."	? American Heart Association suggested: American Heart Association (RRID:SCR_007210. Please make sure this RRID suggestion is accurate before including in your manuscript.

Generated by SciScore.
SciScore only screens the text of the methods section; we apologize if the tool has missed items reported elsewhere in the paper or supplements.

Transparency

?	Open code	If you wrote code to analyze your data, please consider sharing the code in a public repository. This makes it easier for others to reproduce your analyses, and may also aid others seeking to analyze similar datasets.
?	Open data	If permitted, sharing data on a public repository with a persistent identifier (a DOI or accession number) can improve reproducibility and make it easier for other scientists to expand on your work. Papers with open data are cited more often than papers without open data. Some institutions have an expert who can provide advice on data sharing.
✓	Self-acknowledged limitations	"Study Limitations and Future Research"
✓	Conflict of interest statement	Thank you for stating any conflicts of interest.
✓	Funding statement	Thank you for reporting your funding source(s), or stating that there was no specific funding for this work.
n/a	Registration statement	A registered protocol makes the planned research project more transparent and can protect against bias. Please consider registering future confirmatory studies.

Generated by ODDPub, limitation-recognizer, rtransparent, trial-identifier.

Figures

✓	No bar graphs of continuous data	Thank you for not using bar graphs to present continuous data. Many different datasets and data distributions can lead to the same bar graph, and the actual data may suggest different conclusions from the summary statistics alone.
✓	No rainbow color maps	Thank you for not using rainbow colormaps. Rainbow color maps distort readers' perception of the data by introducing visual artifacts and are not accessible to colorblind readers.

Generated by JetFighter, Barzooka.
