# Supplementary material for: Cost impact of chronic care management services in a large multi-specialty practice: A pragmatic outcomes study
Source: PLoS One. 2026 Oct 30;21(10):e0345422. doi: 10.1371/journal.pone.0345422 (PMC13722291; doi:10.1371/journal.pone.0345422)
Supplement: Supplementary file 1 [file pone.0345422.s002.docx]

Reviewer #1: This study is certainly interesting, but some aspects need to be thoroughly reviewed.

Thank you for your thoughtful comments especially on point 2 which made us think more clearly about the thrust of the paper: A real world comparison of typical care vs CCM, not a what if comparison. Our mistake to include the regression results as they distract from the point of the article and would apply only to the specific age/gender mix of a specific population. We also appreciate your very appropriate comments on design but we were extremely limited in the data we could obtain. Below we hope we have addressed your concerns.

1) First, cost estimates should always include drivers that allow for a physical measurement of uptake of resources.

RESPONSE: We assume, but may be wrong, that the reviewer is concerned that reimbursement totals alone can hide whether the differences were due to changes in, say, volume or mix of care. If so, the reviewer is correct.

However, our primary purpose, in line with the budget neutrality constraints imposed on the CMS CMM program, is to compare total reimbursed spending between two groups, we did include Table 2 which summarizes differences in care by place of service which is an important policy marker, typically for substitution effects, of health care costs for this population.

2) Furthermore, the regression analysis is not at all clear.
Why do the authors only control for age and gender? Why do the authors use a linear regression approach? How do we go from estimating the regression coefficients to the results presented in the table? What estimation method was used?

Response: We agree with the reviewer on this point. We pushed a bridge to far. The gist of the paper is about what happened, not what could have happened. We deleted all references to the adjusted results. (As a completely tangential aside, we did do a log transform (The Box Cox was .26) and interpreted the coefficients as multiplicative.)

3) Since this is a case-control study, wouldn't it have been more appropriate to use a difference-in-difference technique?

Response: We could not agree more, but the data for such a comparison were not available.

4) Since this is a cost study, did the authors take into account the typical asymmetry of cost data in their regression analysis? How?

Response: We have deleted the adjusted line in table 1 and any references to them. Also see: Tangential aside in #2 above)

5) Wouldn't it have been appropriate to use a two-step estimation technique? Such as Heckman decomposition, for example?

Response: We agree, if only we had an instrument. Some would say we could use functional form as the instrument but Hay, Leu, and Rohrer (1987) and Manning, Duan, and Rogers (1987) show that’s not usually a good choice.

6) Finally, I completely disagree with the decision not to show the results of the statistical significance tests and find it completely unjustified.

Response: This is now a moot point as we deleted the regressions model but it would be a good side conversation).
