# Supplementary material for: Cost impact of chronic care management services in a large multi-specialty practice: A pragmatic outcomes study
Source: PLoS One. 2026 Oct 30;21(10):e0345422. doi: 10.1371/journal.pone.0345422 (PMC13722291; doi:10.1371/journal.pone.0345422)
Supplement: Supplementary file 2 [file pone.0345422.s003.docx]

Dear Editors of PLOS ONE,

We are pleased with the very positive reviewers of the external reviewers and their conclusion that the manuscript was recommended for publication.

In this version we have rewritten the references in Vancouver style to address the editors concerns.

Sincerely,

Mike Finch, PhD
Corresponding Author
Email: [Mike.Finch@childrensmn.org](mailto:Mike.Finch@childrensmn.org)
