# Supplementary material for: Cost impact of chronic care management services in a large multi-specialty practice: A pragmatic outcomes study
Source: PLoS One. 2026 Oct 30;21(10):e0345422. doi: 10.1371/journal.pone.0345422 (PMC13722291; doi:10.1371/journal.pone.0345422)
Supplement: Peer review history

# Peer review: Decision Letter 0

**DOI:** 10.1371/journal.pone.0345422.r001

**Authors:** Filomena Pietrantonio

21 Apr 2026

Dear Dr. Finch,

- This study is certainly interesting, but some aspects need to be thoroughly reviewed according to the suggestions of the Reviewer 1. In particular it is very important to review cost estimates and the regression analysis. Please answer point by point to the questions of Reviewer 1: First, cost estimates should always include drivers that allow for a physical measurement of uptake of resources. Furthermore, the regression analysis is not at all clear. Why do the authors only control for age and gender? Why do the authors use a linear regression approach? How do we go from estimating the regression coefficients to the results presented in the table? What estimation method was used? Since this is a case-control study, wouldn't it have been more appropriate to use a difference-in-difference technique? Since this is a cost study, did the authors take into account the typical asymmetry of cost data in their regression analysis? How? Wouldn't it have been appropriate to use a two-step estimation technique? Such as Heckman decomposition, for example? Finally, I completely disagree with the decision not to show the results of the statistical significance tests and find it completely unjustified.
- Furthermore, it is absolutely necessary to carefully read the attached report in order to modify the text of the article accordingly
- The decision is justified on PLOS ONE’s publication criteria

plosone@plos.org. When you're ready to submit your revision, log on to https://www.editorialmanager.com/pone/ and select the 'Submissions Needing Revision' folder to locate your manuscript file.

- A letter that responds to each point raised by the academic editor and reviewer(s). You should upload this letter as a separate file labeled 'Response to Reviewers'.
- A marked-up copy of your manuscript that highlights changes made to the original version. You should upload this as a separate file labeled 'Revised Manuscript with Track Changes'.
- An unmarked version of your revised paper without tracked changes. You should upload this as a separate file labeled 'Manuscript'.

If you would like to make changes to your financial disclosure, please include your updated statement in your cover letter. Guidelines for resubmitting your figure files are available below the reviewer comments at the end of this letter.

If applicable, we recommend that you deposit your laboratory protocols in protocols.io to enhance the reproducibility of your results. Protocols.io assigns your protocol its own identifier (DOI) so that it can be cited independently in the future. For instructions see: https://journals.plos.org/plosone/s/submission-guidelines#loc-laboratory-protocols. Additionally, PLOS ONE offers an option for publishing peer-reviewed Lab Protocol articles, which describe protocols hosted on protocols.io. Read more information on sharing protocols at https://plos.org/protocols?utm_medium=editorial-email&utm_source=authorletters&utm_campaign=protocols.

As the corresponding author, your ORCID iD is verified in the submission system and will appear in the published article. PLOS supports the use of ORCID, and we encourage all coauthors to register for an ORCID iD and use it as well. Please encourage your coauthors to verify their ORCID iD within the submission system before final acceptance, as unverified ORCID iDs will not appear in the published article. *Only*  the individual author can complete the verification step; PLOS staff *cannot*  verify ORCID iDs on behalf of authors.

We look forward to receiving your revised manuscript.

Kind regards,

Filomena Pietrantonio

Academic Editor

PLOS One

Journal Requirements:

When submitting your revision, we need you to address these additional requirements.

1. Please ensure that your manuscript meets PLOS ONE's style requirements, including those for file naming. The PLOS ONE style templates can be found at

https://journals.plos.org/plosone/s/file?id=wjVg/PLOSOne_formatting_sample_main_body.pdf and

https://journals.plos.org/plosone/s/file?id=ba62/PLOSOne_formatting_sample_title_authors_affiliations.pdf

2. Thank you for stating the following financial disclosure: [MF consulting fee from WellBox].

Please state what role the funders took in the study.  If the funders had no role, please state: ""The funders had no role in study design, data collection and analysis, decision to publish, or preparation of the manuscript.""

If this statement is not correct you must amend it as needed.

Please include this amended Role of Funder statement in your cover letter; we will change the online submission form on your behalf.

3. Thank you for stating the following in the Financial Disclosure section: [MF consulting fee from WellBox].

We note that one or more of the authors are employed by a commercial company: [Finch & King, Inc]

1. Please provide an amended Funding Statement declaring this commercial affiliation, as well as a statement regarding the Role of Funders in your study. If the funding organization did not play a role in the study design, data collection and analysis, decision to publish, or preparation of the manuscript and only provided financial support in the form of authors' salaries and/or research materials, please review your statements relating to the author contributions, and ensure you have specifically and accurately indicated the role(s) that these authors had in your study. You can update author roles in the Author Contributions section of the online submission form.

Please also include the following statement within your amended Funding Statement.

“The funder provided support in the form of salaries for authors [insert relevant initials], but did not have any additional role in the study design, data collection and analysis, decision to publish, or preparation of the manuscript. The specific roles of these authors are articulated in the ‘author contributions’ section.”

If your commercial affiliation did play a role in your study, please state and explain this role within your updated Funding Statement.

2. Please also provide an updated Competing Interests Statement declaring this commercial affiliation along with any other relevant declarations relating to employment, consultancy, patents, products in development, or marketed products, etc.

Within your Competing Interests Statement, please confirm that this commercial affiliation does not alter your adherence to all PLOS ONE policies on sharing data and materials by including the following statement: ""This does not alter our adherence to  PLOS ONE policies on sharing data and materials.” (as detailed online in our guide for authors http://journals.plos.org/plosone/s/competing-interests) . If this adherence statement is not accurate and  there are restrictions on sharing of data and/or materials, please state these. Please note that we cannot proceed with consideration of your article until this information has been declared.

Please include both an updated Funding Statement and Competing Interests Statement in your cover letter. We will change the online submission form on your behalf.

4. In the online submission form, you indicated that [The data analyzed in this study contain protected health information and are not publicly available. De-identified aggregate data may be made available from the corresponding author upon reasonable request and with permission from the data-holding institution.].

All PLOS journals now require all data underlying the findings described in their manuscript to be freely available to other researchers, either 1. In a public repository, 2. Within the manuscript itself, or 3. Uploaded as supplementary information.

This policy applies to all data except where public deposition would breach compliance with the protocol approved by your research ethics board. If your data cannot be made publicly available for ethical or legal reasons (e.g., public availability would compromise patient privacy), please explain your reasons on resubmission and your exemption request will be escalated for approval.

5. If the reviewer comments include a recommendation to cite specific previously published works, please review and evaluate these publications to determine whether they are relevant and should be cited. There is no requirement to cite these works unless the editor has indicated otherwise.

[Note: HTML markup is below. Please do not edit.]

Reviewers' comments:

Reviewer's Responses to Questions

**Comments to the Author**

1. Is the manuscript technically sound, and do the data support the conclusions?

Reviewer #1: Partly

Reviewer #2: Yes

**********

2. Has the statistical analysis been performed appropriately and rigorously?

Reviewer #1: No

Reviewer #2: Yes

**********

3. Have the authors made all data underlying the findings in their manuscript fully available?

The PLOS Data policy

Reviewer #1: No

Reviewer #2: Yes

**********

4. Is the manuscript presented in an intelligible fashion and written in standard English?

Reviewer #1: Yes

Reviewer #2: Yes

**********

Reviewer #1: This study is certainly interesting, but some aspects need to be thoroughly reviewed.

First, cost estimates should always include drivers that allow for a physical measurement of uptake of resources.

Furthermore, the regression analysis is not at all clear.

Why do the authors only control for age and gender? Why do the authors use a linear regression approach? How do we go from estimating the regression coefficients to the results presented in the table? What estimation method was used?

Since this is a case-control study, wouldn't it have been more appropriate to use a difference-in-difference technique?

Since this is a cost study, did the authors take into account the typical asymmetry of cost data in their regression analysis? How? Wouldn't it have been appropriate to use a two-step estimation technique? Such as Heckman decomposition, for example?

Finally, I completely disagree with the decision not to show the results of the statistical significance tests and find it completely unjustified.

Reviewer #2: Dear Author,

This is an automated report generated for your preprint PONE-D-26-03683. The report is provided by ScreenIT, an independent group of scientists developing automated tools that analyze academic papers.

A set of automated tools screened your manuscript and produced the report below. Each tool was created by your academic colleagues with the goal of supporting authors. The tools check for factors that are important for transparency, rigor, and reproducibility, and we hope that this report will help you improve the reporting in your manuscript.

Within the report, you will find links to more information about the items being checked. These links include helpful papers, websites, or videos explaining why each item is important.

While our screening tools aim to improve and maintain quality standards, they may, on occasion, miss nuances specific to your study or flag something incorrectly. Each tool has limitations, which are described on the ScreenIT website. Please also note that the tools currently screen only the main manuscript file; supplements stored in separate files cannot be analyzed.

ScreenIT is in no way affiliated with medRxiv or bioRxiv. If you would like to provide feedback on the ScreenIT tools, please contact the team at ScreenIT@bih-charite.de.

Thank you for your interest, and best wishes from the ScreenIT team!

**********

what does this mean?). If published, this will include your full peer review and any attached files.

If you choose “no”, your identity will remain anonymous but your review may still be made public.

**Do you want your identity to be public for this peer review?**  For information about this choice, including consent withdrawal, please see our Privacy Policy

Reviewer #1: No

Reviewer #2: No

**********

[NOTE: If reviewer comments were submitted as an attachment file, they will be attached to this email and accessible via the submission site. Please log into your account, locate the manuscript record, and check for the action link "View Attachments". If this link does not appear, there are no attachment files.]

To ensure your figures meet our technical requirements, please review our figure guidelines: https://journals.plos.org/plosone/s/figures

You may also use PLOS’s free figure tool, NAAS, to help you prepare publication quality figures: https://journals.plos.org/plosone/s/figures#loc-tools-for-figure-preparation.

NAAS will assess whether your figures meet our technical requirements by comparing each figure against our figure specifications.

Attachment Submitted filename: report_10.64898+2026.03.07.26347834.pdf

# Author comment: Author response to Decision Letter 1

**DOI:** 10.1371/journal.pone.0345422.r002

22 Jul 2026

Reviewer #1: This study is certainly interesting, but some aspects need to be thoroughly reviewed.

Thank you for your thoughtful comments especially on point 2 which made us think more clearly about the thrust of the paper: A real world comparison of typical care vs CCM, not a what if comparison. Our mistake to include the regression results as they distract from the point of the article and would apply only to the specific age/gender mix of a specific population. We also appreciate your very appropriate comments on design but we were extremely limited in the data we could obtain. Below we hope we have addressed your concerns.

1) First, cost estimates should always include drivers that allow for a physical measurement of uptake of resources.

RESPONSE: We assume, but may be wrong, that the reviewer is concerned that reimbursement totals alone can hide whether the differences were due to changes in, say, volume or mix of care. If so, the reviewer is correct.

However, our primary purpose, in line with the budget neutrality constraints imposed on the CMS CMM program, is to compare total reimbursed spending between two groups, we did include Table 2 which summarizes differences in care by place of service which is an important policy marker, typically for substitution effects, of health care costs for this population.

2) Furthermore, the regression analysis is not at all clear.

Why do the authors only control for age and gender? Why do the authors use a linear regression approach? How do we go from estimating the regression coefficients to the results presented in the table? What estimation method was used?

Response: We agree with the reviewer on this point. We pushed a bridge to far. The gist of the paper is about what happened, not what could have happened. We deleted all references to the adjusted results. (As a completely tangential aside, we did do a log transform (The Box Cox was .26) and interpreted the coefficients as multiplicative.)

3) Since this is a case-control study, wouldn't it have been more appropriate to use a difference-in-difference technique?

Response: We could not agree more, but the data for such a comparison were not available.

4) Since this is a cost study, did the authors take into account the typical asymmetry of cost data in their regression analysis? How?

Response: We have deleted the adjusted line in table 1 and any references to them. Also see: Tangential aside in #2 above)

5) Wouldn't it have been appropriate to use a two-step estimation technique? Such as Heckman decomposition, for example?

Response: We agree, if only we had an instrument. Some would say we could use functional form as the instrument but Hay, Leu, and Rohrer (1987) and Manning, Duan, and Rogers (1987) show that’s not usually a good choice.

6) Finally, I completely disagree with the decision not to show the results of the statistical significance tests and find it completely unjustified.

Response: This is now a moot point as we deleted the regressions model but it would be a good side conversation).

Attachment Submitted filename: Response to Reviewer #1.docx

# Peer review: Decision Letter 1

**DOI:** 10.1371/journal.pone.0345422.r003

**Authors:** Chiara Lazzeri

24 Sep 2026

Dear Dr. Finch,

Thank you for submitting your manuscript to PLOS One. After careful consideration, we feel that it has merit but does not fully meet PLOS One’s publication criteria as it currently stands. Therefore, we invite you to submit a revised version of the manuscript that addresses the points raised during the review process.

Please submit your revised manuscript by Nov 08 2026 11:59PM. If you will need more time than this to complete your revisions, please reply to this message or contact the journal office at plosone@plos.org. When you're ready to submit your revision, log on to https://www.editorialmanager.com/pone/ and select the 'Submissions Needing Revision' folder to locate your manuscript file.

- A letter that responds to each point raised by the academic editor and reviewer(s). You should upload this letter as a separate file labeled 'Response to Reviewers'.
- A marked-up copy of your manuscript that highlights changes made to the original version. You should upload this as a separate file labeled 'Revised Manuscript with Track Changes'.
- An unmarked version of your revised paper without tracked changes. You should upload this as a separate file labeled 'Manuscript'.

If you would like to make changes to your financial disclosure, please include your updated statement in your cover letter. Guidelines for resubmitting your figure files are available below the reviewer comments at the end of this letter.

If applicable, we recommend that you deposit your laboratory protocols in protocols.io to enhance the reproducibility of your results. Protocols.io assigns your protocol its own identifier (DOI) so that it can be cited independently in the future. For instructions see: https://journals.plos.org/plosone/s/submission-guidelines#loc-laboratory-protocols. Additionally, PLOS One offers an option for publishing peer-reviewed Lab Protocol articles, which describe protocols hosted on protocols.io. Read more information on sharing protocols at https://plos.org/protocols?utm_medium=editorial-email&utm_source=authorletters&utm_campaign=protocols.

As the corresponding author, your ORCID iD is verified in the submission system and will appear in the published article. PLOS supports the use of ORCID, and we encourage all coauthors to register for an ORCID iD and use it as well. Please encourage your coauthors to verify their ORCID iD within the submission system before final acceptance, as unverified ORCID iDs will not appear in the published article. *Only*  the individual author can complete the verification step; PLOS staff *cannot*  verify ORCID iDs on behalf of authors.

We look forward to receiving your revised manuscript.

Kind regards,

Chiara Lazzeri

Academic Editor

PLOS One

Journal Requirements:

If the reviewer comments include a recommendation to cite specific previously published works, please review and evaluate these publications to determine whether they are relevant and should be cited. There is no requirement to cite these works unless the editor has indicated otherwise.

[Note: HTML markup is below. Please do not edit.]

Reviewers' comments:

Reviewer's Responses to Questions

**Comments to the Author**

Reviewer #1: (No Response)

Reviewer #3: All comments have been addressed

**********

2. Is the manuscript technically sound, and do the data support the conclusions?

Reviewer #1: Yes

Reviewer #3: Yes

**********

3. Has the statistical analysis been performed appropriately and rigorously?

Reviewer #1: Yes

Reviewer #3: Yes

**********

4. Have the authors made all data underlying the findings in their manuscript fully available?

The PLOS Data policy

Reviewer #1: Yes

Reviewer #3: Yes

**********

5. Is the manuscript presented in an intelligible fashion and written in standard English?

Reviewer #1: Yes

Reviewer #3: Yes

**********

Reviewer #1: Authors have addressed all my concerns and the manuscript is suitable for publication provded that other reviewers agree

Reviewer #3: thank you for your work! I appreciate your collaboration, and in my honest opinion your work is now better than oldest version

**********

what does this mean?). If published, this will include your full peer review and any attached files.

If you choose “no”, your identity will remain anonymous but your review may still be made public.

**Do you want your identity to be public for this peer review?**  For information about this choice, including consent withdrawal, please see our Privacy Policy

Reviewer #1: No

Reviewer #3: No

**********

[NOTE: If reviewer comments were submitted as an attachment file, they will be attached to this email and accessible via the submission site. Please log into your account, locate the manuscript record, and check for the action link "View Attachments". If this link does not appear, there are no attachment files.]

To ensure your figures meet our technical requirements, please review our figure guidelines: https://journals.plos.org/plosone/s/figures

You may also use PLOS’s free figure tool, NAAS, to help you prepare publication quality figures: https://journals.plos.org/plosone/s/figures#loc-tools-for-figure-preparation.

NAAS will assess whether your figures meet our technical requirements by comparing each figure against our figure specifications.

# Author comment: Author response to Decision Letter 2

**DOI:** 10.1371/journal.pone.0345422.r004

8 Oct 2026

The last review was very positive, but the editor posted: After careful consideration, we feel that it has merit but does not fully meet PLOS One’s publication criteria as it currently stands. Is this a rejection? I saw that the references were not in complete Vancouver Style, so I adjusted them in this revision

Attachment Submitted filename: Response to reviewers revised.docx

# Editor report: Decision Letter 2

**DOI:** 10.1371/journal.pone.0345422.r005

**Authors:** Chiara Lazzeri

15 Oct 2026

Cost Impact of Chronic Care Management Services in a Large Multi-Specialty Practice: A Pragmatic Outcomes Study

PONE-D-26-03683R2

Dear Dr. Finch,

We’re pleased to inform you that your manuscript has been judged scientifically suitable for publication and will be formally accepted for publication once it meets all outstanding technical requirements.

Within one week, you’ll receive an e-mail detailing the required amendments. When these have been addressed, you’ll receive a formal acceptance letter and your manuscript will be scheduled for publication.

An invoice will be generated when your article is formally accepted. Please note, if your institution has a publishing partnership with PLOS and your article meets the relevant criteria, all or part of your publication costs will be covered. Please make sure your user information is up-to-date by logging into Editorial Manager at Editorial Manager® and clicking the ‘Update My Information' link at the top of the page. For questions related to billing, please contact billing support.

If your institution or institutions have a press office, please notify them about your upcoming paper to help maximize its impact. If they’ll be preparing press materials, please inform our press team as soon as possible -- no later than 48 hours after receiving the formal acceptance. Your manuscript will remain under strict press embargo until 2 pm Eastern Time on the date of publication. For more information, please contact onepress@plos.org.

Kind regards,

Chiara Lazzeri

Academic Editor

PLOS One

Additional Editor Comments (optional):

Reviewers' comments:

# Editor report: Acceptance letter

**DOI:** 10.1371/journal.pone.0345422.r006

**Authors:** Chiara Lazzeri

PONE-D-26-03683R2

PLOS One

Dear Dr. Finch,

I'm pleased to inform you that your manuscript has been deemed suitable for publication in PLOS One. Congratulations! Your manuscript is now being handed over to our production team.

At this stage, our production department will prepare your paper for publication. This includes ensuring the following:

* All references, tables, and figures are properly cited

* All relevant supporting information is included in the manuscript submission,

* There are no issues that prevent the paper from being properly typeset

You will receive further instructions from the production team, including instructions on how to review your proof when it is ready. Please keep in mind that we are working through a large volume of accepted articles, so please give us a few days to review your paper and let you know the next and final steps.

Lastly, if your institution or institutions have a press office, please let them know about your upcoming paper now to help maximize its impact. If they'll be preparing press materials, please inform our press team within the next 48 hours. Your manuscript will remain under strict press embargo until 2 pm Eastern Time on the date of publication. For more information, please contact onepress@plos.org.

You will receive an invoice from PLOS for your publication fee after your manuscript has reached the completed accept phase. If you receive an email requesting payment before acceptance or for any other service, this may be a phishing scheme. Learn how to identify phishing emails and protect your accounts at https://explore.plos.org/phishing.

If we can help with anything else, please email us at customercare@plos.org.

Thank you for submitting your work to PLOS One and supporting open access.

Kind regards,

PLOS One Editorial Office Staff

on behalf of

Dr. Chiara Lazzeri

Academic Editor

PLOS One
